# SERUM FACTORS DRIVE DIFFERENCES IN MITOCHONDRIAL BIOENERGETICS ASSOCIATED WITH HUMAN AGING

**DOI:** 10.1093/geroni/igae098.1871

**Published:** 2024-12-31

**Authors:** Stephanie Heimler, Brian Chen, Toshiko Tanaka, Ann Zenobia Moore, Jaclyn Bergstrom, Luigi Ferrucci, Anthony Molina

**Affiliations:** University of California San Diego, La Jolla, California, United States; University of California San Diego, La Jolla, California, United States; National Institute on Aging, Baltimore, Maryland, United States; National Institute on Aging, Baltimore, Maryland, United States; UC San Diego, La Jolla, California, United States; National Institute on Aging, Baltimore, Maryland, United States; University of California San Diego, La Jolla, California, United States

## Abstract

Heterochronic parabiosis studies in mice have demonstrated that circulating factors in blood drive multiple features of aging, including mitochondrial bioenergetic decline. To determine if circulating factors in human blood can drive age-related bioenergetic decline, we treated fibroblasts from younger (33y.o.) and older (72y.o.) adult donors with diluted serum samples representing the adult human life-course (22-92y.o). The GESTALT (Genetic and Epigenetic Signatures of Translational Aging Laboratory Testing) study provided serum samples from 88 healthy participants free from major chronic conditions. We show that the maximal bioenergetic capacity of both younger and older fibroblasts treated with serum for 24 hours negatively correlates with the chronological age of the serum donor (R=-0.28, P=0.01; R=-0.25, P=0.02, respectively). To test the hypothesis that the bioenergetic effects of serum are more closely associated with biological age than chronological age, we used DNA methylation data to calculate first and second generation epigenetic clocks (Horvath, Hannum, PhenoAge, and GrimAge). Our results indicate that the bioenergetic effects of serum are most closely related to second generation clocks, which are trained to capture age-related phenotypes and mortality risk. For example, the effects of serum on fibroblasts derived from younger and older individuals were most closely related to GrimAge (R=-0.36, P< 0.01; R=-0.31, P< 0.01, respectively). These results suggest that the bioenergetic effects of circulating factors may explain the heterogeneity of biological aging among participants of the same chronological age. Ongoing studies are pairing our high-throughput respirometric analyses with multi-omic data to identify molecules that directly impact mitochondrial bioenergetic decline with advancing age.

